# Semi-automatic robotic puncture system based on deformable soft tissue point cloud registration

**DOI:** 10.1007/s11548-024-03247-3

**Published:** 2024-10-26

**Authors:** Bo Zhang, Kui Chen, Yuhang Yao, Bo Wu, Qiang Li, Zheming Zhang, Peihua Fan, Wei Wang, Manxia Lin, Masakatsu G. Fujie

**Affiliations:** 1 WuXi AMIT Intelligent Medical Technology Col., Ltd., Wuxi, 214000 China; 2 Future Robotics Organization, Waseda University, Tokyo, 1620044 Japan; 3 The First Affiliated Hospital of Sun Yat-sen University, Guangzhou, 510000 China

**Keywords:** Puncture system, Surgical robot, Image recognition, Three-dimensional reconstruction, Point cloud registration

## Abstract

**Purpose:**

Traditional surgical puncture robot systems based on computed tomography (CT) and infrared camera guidance have natural disadvantages for puncture of deformable soft tissues such as the liver. Liver movement and deformation caused by breathing are difficult to accurately assess and compensate by current technical solutions. We propose a semi-automatic robotic puncture system based on real-time ultrasound images to solve this problem.

**Method:**

Real-time ultrasound images and their spatial position information can be obtained by robot in this system. By recognizing target tissue in these ultrasound images and using reconstruction algorithm, 3D real-time ultrasound tissue point cloud can be constructed. Point cloud of the target tissue in the CT image can be obtained by using developed software. Through the point cloud registration method based on feature points, two point clouds above are registered. The puncture target will be automatically positioned, then robot quickly carries the puncture guide mechanism to the puncture site and guides the puncture. It takes about just tens of seconds from the start of image acquisition to completion of needle insertion. Patient can be controlled by a ventilator to temporarily stop breathing, and patient’s breathing state does not need to be the same as taking CT scan.

**Results:**

The average operation time of 24 phantom experiments is 64.5 s, and the average error between the needle tip and the target point after puncture is 0.8 mm. Two animal puncture surgeries were performed, and the results indicated that the puncture errors of these two experiments are 1.76 mm and 1.81 mm, respectively.

**Conclusion:**

Robot system can effectively carry out and implement liver tissue puncture surgery, and the success rate of phantom experiments and experiments is 100%. It also shows that the puncture robot system has high puncture accuracy, short operation time, and great clinical value.

## Introduction

With the development of robotic technology, a variety of minimally invasive, high-precision surgical robot systems or solutions have been developed [1–3]. In the field of liver tumor treatment, due to respiration, the position of the liver in the abdominal cavity and the shape of the liver itself will change at any time. It is estimated that the displacement of liver along the head and feet of the human body can reach about 4 cm [4] during breathing. How to accurately locate the tumor when the liver is deformed and displaced is a difficulty for liver surgery robots.

Some robots were invented to solve the problem of accurate puncture of liver tumors. EPIONE robotic system developed by Quantum Surgical, can be used for multiple kinds of soft tissue puncture surgeries including liver. The system uses a device called patient reference fixed on the surface of patient body to register the patient’s position and CT images to locate the puncture target [5, 6]. A respiratory monitoring module is used to monitor human respiratory and indirectly correction registration and puncture guidance. As there is no guarantee that patients have the same breathing state at CT scan and surgery, it is quite difficult to directly locate the position of the tumor inside the human body based on the mark fixed on human surface. Although there is a respiratory module to predict the deformation of the liver, this indirect method cannot ensure the accuracy of prediction. Doctors sometimes need multiple CT scans to confirm the puncture results, which also increases the patient’s radiation dose [7].

We proposed a precise positioning and guidance puncture system based on real-time ultrasound images, to solve the problem of difficulty in assessing liver movement and deformation during puncture surgery. The patient only needs to stop breathing for a short period of time, and the system can quickly complete the registration of the current patient position with the CT images. Ultrasound is then used to guide puncture in real time, which not only improves puncture accuracy but also greatly reduces the patient’s radiation dose.

### System design

This robot system consists of two parts, preoperative planning and intraoperative navigation. The preoperative planning software enables to review CT images, segment CT blood vessels, and plan needle insertion trajectory. It is worth noting that the liver vessels are not clear in plain CT, and contrast liver CT is required for preoperative planning. While the intraoperative navigation system assists doctors in completing surgeries. There are three key technologies in intraoperative navigation system, namely, 1. real-time cloud point reconstruction, 2. point cloud registration based on feature points, and 3. accurate puncture guide mechanism.

Real-time cloud point reconstruction module can realize the rapid 3D point cloud reconstruction of the current patient’s liver blood vessels, providing a basis for registration with CT data. Point cloud registration based on feature points module is a method that considers the structural characteristics of blood vessels and allows two deformed blood vessels to accurately match each other. In this system, feature points refer to the intersections of blood vessels, where the normal vectors on these points change greatly. After the registration is completed, the puncture target is automatically determined, the puncture guide mechanism can help doctors to quickly complete needle insertion. In the whole process, ultrasound and CT fusion images are provided, which are very useful reference information for doctors [8, 9]. All these steps are fast, totally taking tens of seconds, and the patient only needs to hold his breath briefly. Therefore, this system can eliminate the influence of liver deformation caused by breathing and accurately complete needle insertion.

**Fig. 1:**
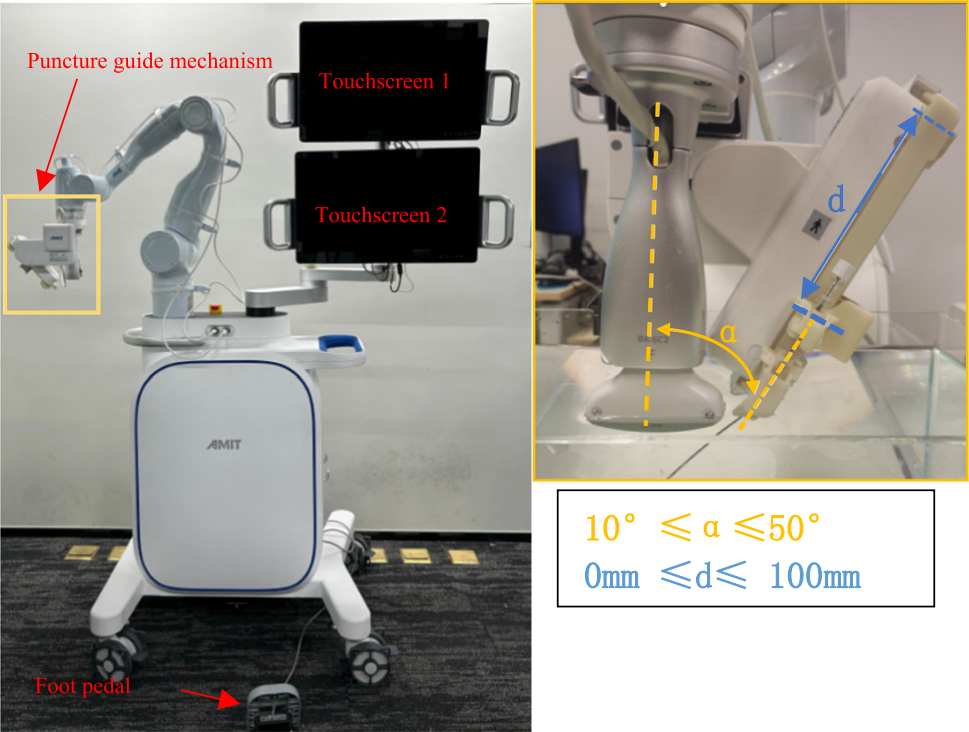
System structure

The main parameters of this designed robot system are shown in Table 1. Figure 1 shows the overall appearance of this system and designed puncture guide mechanism.

**Table 1:** System parameters

System	Item	Specification_
Total	Length × width × height (mm)	900 × 900 × 1950
	Weight (kg)	300
	Degree of freedom	9
	Maximum external contact force (N)	65
Robotic arm	Robot	Agile robots Diana 7 Med × 1
	Degrees of freedom	Total 7
	Maximum load (kg)	7
	Positioning accuracy (mm)	0.5
	Repeat positioning accuracy (mm)	0.1
Puncture guide mechanism	Degrees of freedom	Total 2
		Rotational DOF × 1
		Translational DOF × 1
	Puncture angle (degrees)	10–50
	Puncture depth (mm)	0–100
	Puncture accuracy (mm)	Less than 0.6
Sensor	Six-axis force torque sensor	Kunwei KWR75B × 1
	*Fx*, *Fy*, *Fz* (N)	± 200
	*Mx*, *My*, *Mz* (Nm)	± 8
	Accuracy (%FS)	0.5
Operation	Touchscreen	2
	Foot pedal	1

## Method

This system can still accurately locate liver tumor, even if breathing has caused the liver’s position to be different from when the CT was taken. These three key technologies mentioned above can be used to achieve this goal.

### Real-time cloud point reconstruction of liver blood vessels

Identifying and puncturing liver tumors directly in ultrasound images is the most direct and effective surgical option. However, liver tumors may not be clear on ultrasound images and have various shapes, and it is difficult to accurately identify all kinds of liver tumors by one model or algorithm. Blood vessels inside the liver are abundant and have clear outlines in ultrasound image, making them easy to accurately identify. Breathing affects the position of the liver in the abdominal cavity and can cause deformation of both blood vessels and tumors. Tumors and blood vessels are fixed inside the liver, and their relative positional relationship is unchanged. Therefore, easily identifiable vascular structures under ultrasound can be used to accurately locate tumors.

**Fig. 2:**
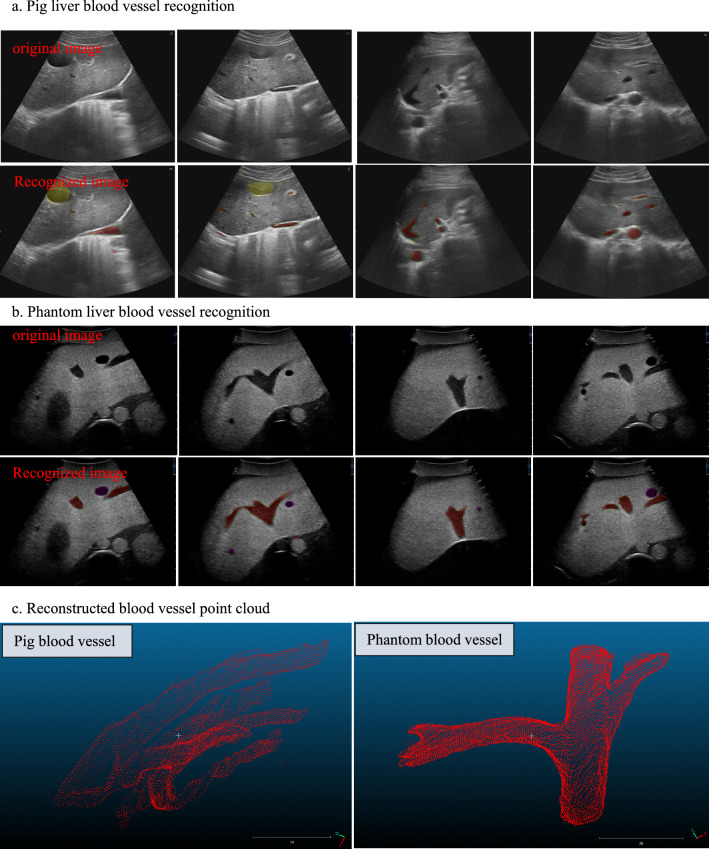
Liver blood vessel identification results

In ultrasound images, blood vessels will appear in a hypoechoic state, with obvious grayscale differences between the blood vessel wall and surrounding tissue. Blood vessels in the liver can be identified by a variety of methods [10–12]. To reduce misidentification, we use improved U-Net model and add preprocessing during input, data filtering, and judgment during output. Only classes with a confidence level above 0.5 are considered correct outputs, and the others are considered background. In addition, continuous regions with a number of pixels less than 40 or greater than 50,000 are also considered misidentifications. Using this method, we obtained two AI models for phantom and pig liver blood vessel recognition using the training set and validation set as shown in Table 2. The test results of the two models on the test set show that the average recognition Intersection over Union (IOU) of pig liver vessels and phantom vessels were 0.73 and 0.82. In Fig. 2, the green contour lines (width about 1 pixel) are annotation results of blood vessels and considered ground true. The red masks are the recognition results of blood vessels. The contour lines and masks of other colors represent the recognition results of other tissues (the yellow area represents the gallbladder, and the purple area represents the tumor).

**Table 2:** Training data and test results of two models

Blood vessel	Training set	Validation set	Test set	Average IOU of test set
Pig liver	1450	307	306	0.73
Phantom liver	1120	192	200	0.82

The identification of liver blood vessels is the basis for further reconstruction of blood vessels point cloud. In this robotic system, the ultrasound probe is rigidly attached to the end of the robotic arm, and robot arm has the function to get the positional information of ultrasound probe. Therefore, the spatial position information of all ultrasound images acquired by this system can be obtained. Briefly, the point cloud reconstruction steps: identify the blood vessels in the ultrasound image, obtain the real-time positional information of ultrasound images, and then construct point cloud of liver blood vessels according to the reconstruction algorithm.

### Point cloud registration based on feature points

The liver is rich in blood vessel, each part of the blood vessel has obvious characteristics and is easy to identify. There are different methods to obtain blood vessel point clouds from CT data [13–15]. We developed preoperative planning software based on the Medical Imaging Interaction Toolkit (MITK) for CT data segmentation and needle insertion trajectory planning.

**Fig. 3:**
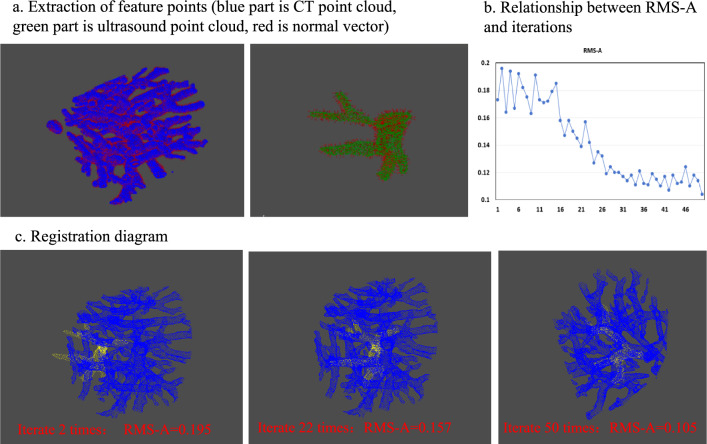
Schematic of point cloud registration based on feature points

A feature point-based registration method based on improved ICP algorithm [16] is used in this system. The first step is to calculate feature points from CT point cloud and ultrasound point cloud. The root-mean-square average distance (RMS-A) of paired feature points is used for registration evaluation. This method not only fully considers the characteristics of liver blood vessels, making tumor prediction more accurate, but also reduces the number of calculation points, speeds up the calculation, and improves registration efficiency. The efficiency of this method is more than twice that of traditional solutions, and the average distance of the feature point groups after registration is about 0.1 mm. Figure 3b shows the extraction of registration feature points and the registration process of point clouds. After finishing the registration, the system will automatically calculate the CT slices that match the current ultrasound image based on the registration matrix. Then, the CT slices are scaled according to the proportion of the ultrasound image and fused with the current ultrasound image.

### Accurate puncture guide mechanism

**Fig. 4:**
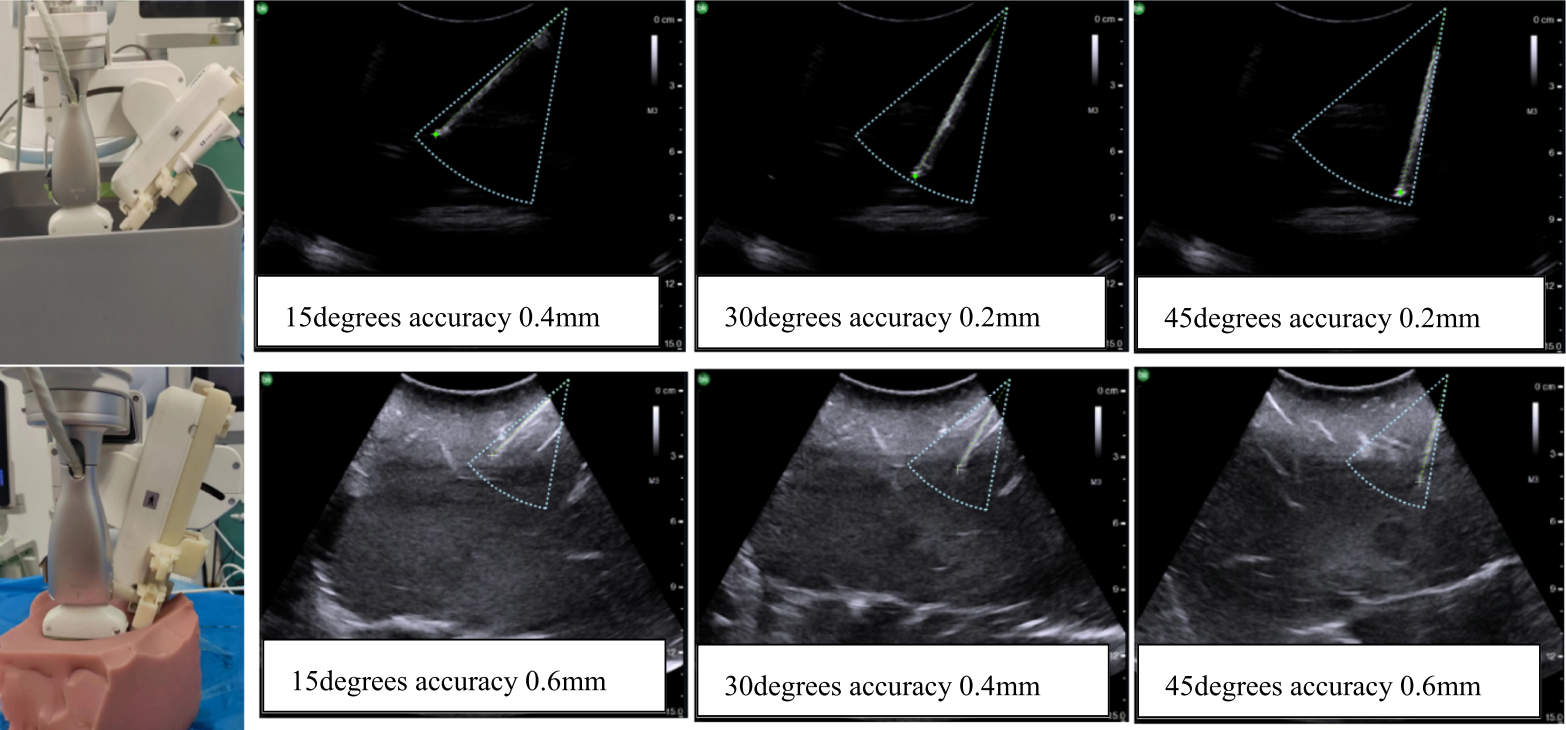
Navigation accuracy of puncture guidance mechanism

After the above registration and fusion process are completed, robot arm carries puncture guide mechanism to the planned puncture site. The accuracy of the puncture guide mechanism will determine the success of puncture surgery. To ensure the rigidity, accuracy, and stability of it, we abandoned the traditional parallel multi-link structure and adopted a customized design based on high-strength arc guide rails, high-strength linear guide rails, and high-precision servo motors. The theoretical control accuracy of the needle tip position can reach 0.1 mm by using this design. Since the dimensions of each component of the puncture guide mechanism are known and the installation position of the ultrasound probe is fixed, the pre-pass trajectory of the puncture needle can be predicted based on the puncture angle and depth. The pre-pass trajectory the needle is marked in green dotted line. Figure 4 shows the accuracy test of puncture guide mechanism, the results indicate that the average puncture accuracy of it in water (the deviation between the needle tip and the end of the guide wire) is within 0.4 mm, and the average puncture accuracy in silicone tissue (used to simulate human body) is within 0.6 mm.

## Experiments

**Fig. 5:**
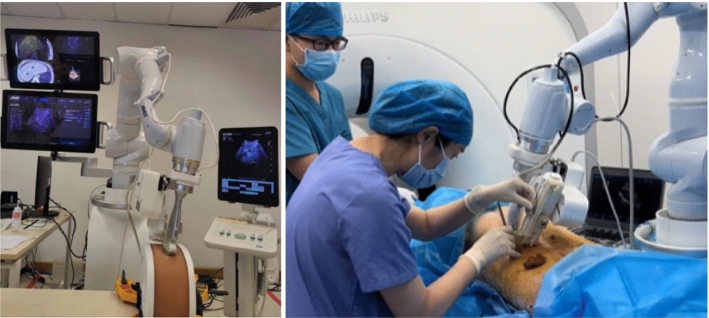
Phantom and animal experimental

Phantom and animal experiments were conducted to test the reliability and accuracy of this robotic puncture system. The on-site settings of these two experiments are shown in Fig. 5.

### Experimental process

These two experiments mainly include the following steps:

#### Experiment preparation

To accurately evaluate the puncture accuracy, definite puncture target points are needed. Titanium particle has good display effects under both ultrasound and CT. Two metal titanium particles (diameter 1 mm and length 2 mm) were implanted into the pig liver as puncture targets.

A phantom (CRIS 057A) contains models of blood vessels, bones, and tumors which was used in this experiment. Four tumors were selected as puncture targets.

#### CT scan

CT scans were performed on phantom and pig. When scanning pig liver CT, contrast agent needs to be injected to show the blood vessels clearly. For phantoms, ordinary CT scan is sufficient.

#### Robotic needle insertion planning

Preoperative planning software was used to segment the blood vessels from CT and convert them into point cloud. In phantom experiments, six different puncture trajectories for each tumor in phantom and one puncture trajectory for each titanium particle in pig liver were designed.

#### Robotic-guided needle insertion

Place ultrasound probe on the body surface where the blood vessels are clear around puncture targe. For animal experiments, the breathing of pigs under general anesthesia needs to be stopped (by stopping the ventilator). Robotic arm then drove the ultrasound probe to scan part of the liver and construct a 3D point cloud of liver blood vessels. Using feature point-based registration algorithm, robot system automatically registered the reconstructed vascular ultrasound point cloud with the CT point cloud. The ultrasound image and the corresponding CT image are automatically fused according to the registration results. Then, system will automatically control the robotic arm to take the probe and puncture guide mechanism to the target position.

A 14-gauge ablation needle (KY-2450KBY-2450B, Canyon Medical, China) was used in the phantom experiment, and a 14-gauge trocar (XD 2.1*200, Zibo Mingyuan Industry, China) was used to implant a titanium particle into pig liver as a test point. Then, ventilator was turned on again to restore the pig’s breathing. It should be noted that in pig puncture surgery, a scalpel is needed to cut the skin at the needle entry point to facilitate the penetration of the puncture needle.

#### Puncture accuracy measurement

**Fig. 6:**
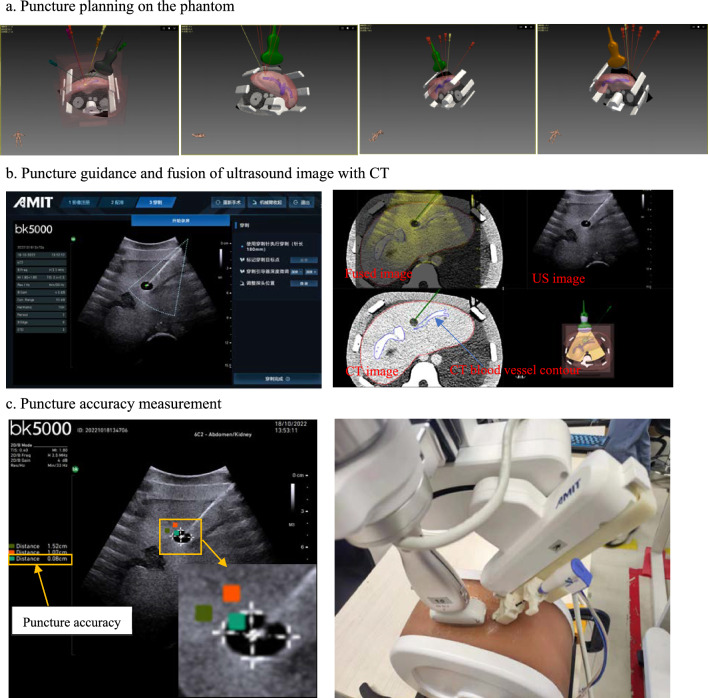
Schematic diagram of phantom experiment

In phantom experiments, the puncture target point can be considered as the intersection point of the four points on the edge of the tumor (the uppermost, the lowermost, the leftmost, and rightmost points) in the ultrasound image, as shown in Fig. 6c. The distance between the puncture target point and the puncture needle tip was measured directly from ultrasound. Since there were 24 phantom experiments in total, steps D and H were repeated 24 times.

In animal experiments, after implanting the first target, step D was repeated one time to puncture the second target and implant the second titanium particle as test point. MITK was used to measure the distance between test points and target points in CT images.

## Results

### Phantom experimental results

The phantom experimental results are shown in Fig. 6. Figure 6a shows puncture target points and puncture trajectories in preoperative planning. There are four puncture target points and 24 puncture trajectories in total. Figure 6b indicates CT and ultrasound image fusion results after registration. The green dotted line in ultrasound image is virtual puncture guide line. Figure 6c shows the error test between the needle tip and the puncture target. In this puncture experiment, the puncture accuracy is 0.08 cm.

The results of the 24 experiments on the phantom are shown in Table 3. As shown from Table 3, the operation time of step D in each experiment is relatively stable, and the average time is 64.6 s (maximum 67 s and minimum 62 s). In terms of puncture accuracy, the average puncture error between the puncture needle tip and the target center is 0.8 mm, and the maximum and minimum errors are 1.1 mm and 0.6 mm, respectively. The phantom experiment results show that the accuracy of this system can meet the designed target (within 3 mm), and the operation time can meet the requirements of breath holding for animal and human surgery.

**Table 3:** Operation time and puncture accuracy of phantom experiments

	Experiments	1	2	3	4	5	6
Tumor 1	Operation time (s)	64	63	65	62	64	63
	Puncture accuracy (mm)	0.8	0.6	0.7	0.9	1.0	0.8
Tumor 2	Operation time (s)	65	67	64	66	65	63
	Puncture accuracy (mm)	1.1	0.8	0.7	0.9	0.6	0.8
Tumor 3	Operation time (s)	63	64	63	65	65	67
	Puncture accuracy (mm)	0.6	0.9	0.8	1.0	0.8	0.8
Tumor 4	Operation time (s)	67	66	64	67	65	64
	Puncture accuracy (mm)	0.6	0.7	0.9	0.8	0.7	0.9

### Animal experimental results

**Fig. 7:**
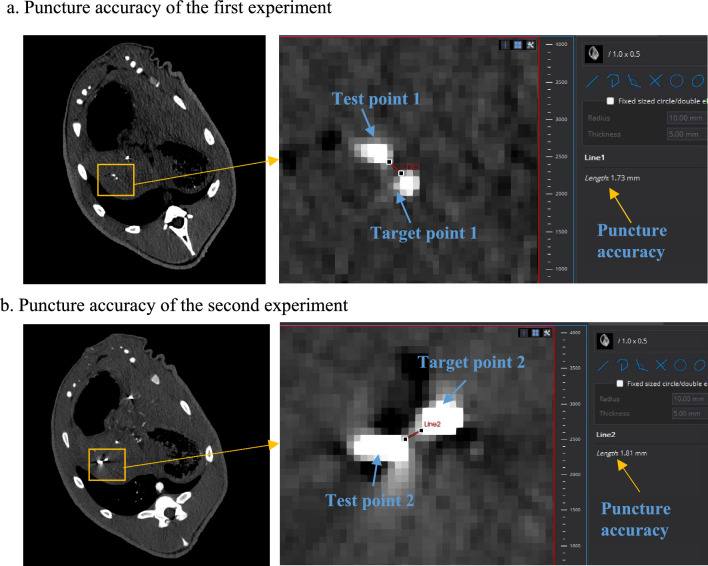
Puncture accuracy in animal experiments

In this animal experiment, two puncture surgeries were conducted, and two titanium particles were implanted as test points. MITK is used to read CT data and evaluate puncture errors. The test results of the two experiments are shown in Fig. 7. In the first experiment, the distance between the test point and the target point is 1.73 mm, while in the second experiment, the distance between two points is 1.81 mm.

## Discussion

In phantom and animal experiments, this designed robotic system ran smoothly. No misoperation occurred, and no adverse events occurred during experiments. Experiment results show that this system has good safety and reliability.

In terms of surgical efficiency, the operation time of the semi-automatic robotic puncture system is stable, and phantom experiments show that the average operation time is 64.6 s. More than 90% of the surgical process is completed autonomously by robots. The system has a high degree of automation and relies less on the doctor’s experience, allowing doctors to concentrate more on controlling the effects of surgery. During the surgery, there are only two places that require doctor’s manual intervention. The first one is to find a place with clear blood vessels and put ultrasound probe on it. The second one is to confirm that the puncture navigation is correct and insert puncture needle along the puncture guide mechanism. For the first one, because sonographers are remarkably familiar with the conditions of liver blood vessels, they can easily complete the placement of ultrasound probe. For the second point, because of the auxiliary holding function of the puncture guide mechanism, the operator does not need to worry about the puncture needle deviating from the ultrasound plane and the preset trajectory. If the doctor confirms the puncture path is suitable, it only takes a few seconds to complete the puncture action.

In terms of accuracy, in the phantom experiment, the distance between puncture needle tip and the puncture target was distributed between 0.6 and 1.1 mm, with an average of 0.8 mm. Animal experiment results show that the minimum distances between the test points and the target points are 1.73 mm and 1.81 mm, respectively, which can meet the design requirements.

It has two reasons leading to the above test results. The first reason is that the system’s has three technical key points and can accurately locate tumor targets. Another reason is that during the puncture process, the puncture guide mechanism can provide stable assistance, automatically adjust its puncture depth and angle, doctors can quickly complete the procedure. Due to the 14-gauge needle has good rigidity and the needle insertion speed is fast, the deformation and trajectory deviation of the needle during the puncture process is small [17].

The puncture accuracy of the tumor in this system depends on the relative position to the blood vessels. When the tumor is far away from the main identifiable blood vessels, the puncture accuracy of the tumor may become poor. Surgery is recommended for tumors that are within 3 cm of a major blood vessel. If the registration fails after repeated attempts, the doctor can switch to manual mode. In this mode, the doctor can hold the ultrasound probe to find the puncture position and manually adjust the puncture angle and depth. The system provides a puncture guide line to assist the doctor in completing the puncture operation.

## Conclusion

To solve the problem of difficulty in locating intrahepatic tumors due to liver deformation caused by breathing. This paper proposes a registration method based on real-time ultrasound and feature points. Through preoperative planning software and intraoperative navigation system, high-precision puncture surgery can be realized. Three key technologies in intraoperative navigation system are described in detail, and quantitative experimental results are given in method part. Phantom and animal experiments were conducted, and the experimental results prove the stability and puncture accuracy of the proposed system. In the next step, we will conduct clinical trials, and there are two problems that need to be confirmed and resolved. The first one is how to ensure the ultrasound probe will not press on the ribs after reaching the planned target position. The second one is how to automatically adjust the position of the ultrasound probe after detecting that the probe position is inappropriate. The current registration method used is the rigid registration. In the future, deformable registration method is also a direction that needs to be studied.
